# The Effect of Alcohol‐Based Virucidal Hand Sanitizers on Skin Barrier Function—A Randomised Experimental Study

**DOI:** 10.1111/cod.14808

**Published:** 2025-05-12

**Authors:** Michal Gina, Robert Ofenloch, Ingeborg Schwebke, Nils‐Olaf Hübner, Thomas Brüning, Manigé Fartasch

**Affiliations:** ^1^ Institute for Prevention and Occupational Medicine of the German Social Accident Insurance (IPA) Institute of the Ruhr University Bochum Bochum Germany; ^2^ Occupational Dermatology, Department of Dermatology Ruprecht‐Karls University Heidelberg Germany; ^3^ Expert Committee on Virus Disinfection of the German Association for the Control of Viral Diseases (DVV) e.V. And the Society for Virology (GfV) e.V. Heidelberg Germany; ^4^ Institute of Hygiene and Environmental Medicine University Medicine Greifswald Greifswald Germany

**Keywords:** alcohol‐based hand rubs, barrier disturbance, irritant contact dermatitis virucidal activity, occupational, skin barrier, non‐invasive measuring methods

## Abstract

**Background:**

Applying alcohol‐based hand rubs (ABHRs) is a proven means of combating hand‐borne microorganisms. In addition to their bactericidal activity, some rubs also have virucidal properties (ABVHRs). Frequent use of ABHRs can result in skin irritation.

**Objectives:**

This study investigates the impact of four commercially available ABVHRs on skin function (ABVHR A‐D). ABVHR‐A and ABVHR‐B contained ethanol in higher concentrations, whereas ABVHR‐C and ABVHR‐D comprised ethanol and 1‐propanol at lower concentrations combined with phosphoric acid (PA).

**Methods:**

Using occlusion‐modified tandem irritation tests and standard bioengineering methods, we assessed the effects of these ABVHRs and controls (ethanol, isopropanol, PA, water) on 48 healthy Caucasian volunteers' skin.

**Results:**

In general, alcohols and ABVHRs were well tolerated. However, the results revealed significant changes in transepidermal water loss (TEWL), corneometry, and colorimetry between baseline and day 3 for all ABVHRs and controls, particularly for ABVHR‐D (TEWL change 6.43 (SD 1.40) to 8.76 (SD 3.87)). Although the differences between the ABVHRs were not statistically significant, ABVHR‐A and ABVHR‐D significantly increased TEWL compared to water. Most ABVHRs demonstrated a better skin irritation profile than pure ethanol (80%) and isopropanol (70%). PA slightly reduced corneometry values.

**Conclusions:**

This study suggests that the irritative potential of ABVHRs varies, likely due to differences in alcohol type (1‐propanol in particular) and concentration. At the tested concentration, PA appears to be well tolerated and may enhance virucidal activity without significantly increasing skin irritation.

## Graphical abstract

Our study suggests that the irritative potential of alcohol‐based virucidal hand rubs (ABVHRs) varies, likely due to differences in alcohol type (1‐propanol in particular) and to the proportions and concentrations of alcohols or alcohol mixtures used. PA appears to be well tolerated and may enhance virucidal activity.

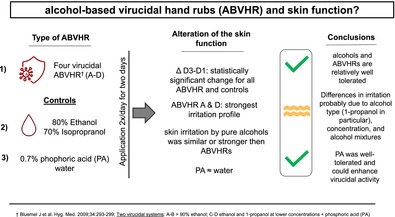

## 1 Introduction

Frequent and prolonged exposure to water, detergents, and sanitizers in conjunction with glove occlusion appears to be one of the major etiological factors responsible for the development of occupational irritant contact dermatitis affecting hands [1, 2, 3, 4, 5]. Studies comparing the cumulative irritative effects of repeated exposure to alcohol‐based hand rubs (ABHRs) versus water and soap/detergents [5] have demonstrated that detergents have a more pronounced skin‐damaging effect. In contrast, alcohol exposure only mildly affects the skin barrier and is generally well tolerated [6, 7, 8]. The alcohols which are used in hand rubs are categorised as primary, secondary, and tertiary alcohols, depending on how many carbon atoms are linked to the carbon atom bearing the hydroxyl group [9]. Ethanol and 1‐propanol (n‐propanol) are examples of primary alcohols, while 2‐propanol (synonym: isopropyl alcohol, CAS 67‐63‐0, isopropanol) is a secondary alcohol and a structural isomer of 1‐propanol. The experimental real‐life scenario study of Conrad et al. demonstrated that virucidal ABHRs differ in their irritative properties and could affect skin barrier function [10]. However, this effect was not observed in epidemiological studies (e.g., Hamnerius et al. [11] and Visser et al. [12]) showing an association between prevalence/incidence of hand eczema and hand washing frequency, whereas no such link has been found between frequency of hand disinfection and prevalence/incidence of hand eczema.

Unfortunately, the available information on the irritative potential of products is rather sparse. Recent studies indicate that some ingredients, for instance n‐propanol, might be more irritative, especially on atopic skin [13]. Moreover, some studies have reported that the effect of alcohols on barrier lipids is inversely related to their concentration [14].

Viral epidemics, such as the COVID‐19 pandemic, have resulted in stricter hygienic precautions, causing an increase in hand eczema not only among healthcare workers [15] but also in general population [16, 17]. Some enveloped viruses, such as the severe acute respiratory syndrome coronavirus 2 (SARS‐CoV‐2), can be inactivated by alcohol concentrations of 70%–80% or even lower [18]. The labels of alcohol‐based virucidal handrubs (ABVHRs) indicate which viruses they are effective against, for example “limited spectrum of virucidal activity” (inactivation of adenovirus, norovirus, rotavirus, and enveloped viruses) or “virucidal activity” (additionally inactivating poliovirus, for example). Typically, higher alcohol concentrations are required for virucidal activity [19, 20, 21] In addition, virucidal systems based on a combination of different alcohols at lower concentrations with active ingredients such as phosphoric acid (PA) can be employed [22]. However, some reports suggest that PA causes increased skin irritation [10, 23, 24]. In any case, both bactericidal and yeasticidal activity are prerequisites for every ABHR [25, 26].

The present experimental study employed an occlusion‐modified tandem irritation test to investigate the influence of four commercially prepared ABVHRs on the skin of healthy volunteers. The ABVHRs contained differing concentrations and compositions of various alcohols (ethanol, 1‐propanol) and emollients, either with or without PA.

## 2 Material and Methods

### 2.1 Characterisation of Participants

Forty‐eight healthy adult volunteers with no history of previous or ongoing skin or systemic diseases were included in the study. The participants were informed in detail about all procedures and potential side effects. They were not permitted to use detergents, emollients, or moisturisers during the study period. Exclusion criteria were endocrine or immune system disease, pregnancy or breastfeeding, treatment with drugs such as immunosuppressants, and exposure to UV radiation within the preceding four weeks.

All tests were performed in compliance with medical research ethical requirements, the Declaration of Helsinki, and with approval from the local ethics committee (Medical Faculty, Ruhr‐University of Bochum, Reg. No: 2015–5257). Written consent was obtained from all participants. Participants were examined at the Institute for Prevention and Occupational Medicine (IPA) in Bochum from April to September 2015.

### 2.2 Study Design

#### 2.2.1 Selection of Virucidal Alcoholic Hand Rubs

In Germany, ABHRs used for disease prevention in the medical sector must have demonstrated antimicrobial activity as per European standards [27, 28, 29]. Additionally, ABVHRs must also have demonstrated activity against viruses [30] in accordance with the guidelines of the German Association for the Control of Virus Diseases (DVV)/Robert‐Koch‐Institute (RKI—German Institute of Public Health) or the European Standard [31, 32].

Official ABVHR labelling lists from the Association for Applied Hygiene [33] (VAH list from 01 January 2014), the 2013 RKI list and additional safety data sheets/product information were revised with respect to labelling ‘virucidal activity’, ingredients and alcohol concentrations [34]. Commercially available ABVHRs were selected in cooperation with the Central Unit for Infection Prevention and Control, Greifswald University Medical Center, Greifswald, Germany (Prof. Hübner), and the RKI (Dr. Schwebke). Selection criteria were (a) labelling ‘virucidal activity’ and (b) containing one of two antimicrobial systems, either with high‐concentrated ethanol without the addition of PA or a mix of lower‐concentrated alcohols (ethanol, 1‐propanol) with PA. All four ABVHRs are labelled as having ‘virucidal activity’ in accordance with the criteria set by the DVV and the RKI guidelines published in 2008 [35].

Two products containing PA (ABVHR‐C and ‐D) at a low concentration similar to that of a selected control [22], and two containing only alcohols were selected (Table 1). All the ABVHRs contained emollients and humectant substances (e.g., glycerin, amount not specified by the manufacturer).

**TABLE 1:** The composition of selected virucidal alcohol‐based hand rubs (ABVHRs).

	Active agent/(gramm)	Concentration (v/v%)^a^	Additional ingredients/moisturisers	Declared application time for virucidal activity^b^
A	Ethanol (95 g)	< 100%	0.5 g glycerol, 2‐butanol, 1‐tertradicanol	2 min
B	Ethanol (89 g)	< 90%	Methylethylketon, macrogol‐6‐gycerolcaprylocaprat, lactic acid, fragrance (orange, limonen), butan‐2‐on	^c^2 min
C	Ethanol (57.6 g) 1‐Propanol (10 g)	< 55% < 15%	Propylenglycol, glycerol, butan‐1,3‐diol, lanolinpolyoxyethylen, fragrance, 2‐butanon, PA	1 min
D	Ethanol (45 g) 1‐Propanol (18 g)	< 55% < 25%	Macrogol 4000, butan‐2‐on, octyldodecanol, glycerol, PA	1 min

^a^ Concentration as per safety data sheets (calculated actual value see in text).

^b^ Recommended application time to achieve virucidal activity according to the RKI 2013.

^c^ ABVHR‐B according to the manufacturer.

The safety data sheets and product information documents for the ABVHRs were not standardised. However, by combining all the provided information, we were able to calculate the alcohol concentrations in v/v% (in brackets as follows: ABVHR‐A: 95 g/100 g of ethanol 99%) (actual: 94.97%), ABVHR‐B: 89 g/100 g of ethanol 96% (actual: 89.2%); ABVHR‐C: 57.6 g/100 g of ethanol 96% (actual: 58.59%), 1‐propanol 10 g (actual: 10.2%), ABVHR‐D: 45 g/100 mL of ethanol 99% (actual: 56.68%), 1‐propanol 18 g/100 mL (actual: 22.5%).

After evaluation of ABVHR lists published by the RKI, the following controls were selected: ethanol 80% v/v, 2‐propanol (isopropanol) 70% v/v, water, and PA in aqueous solution 0.7% g/g [22]. The alcohol concentrations of the ethanol and 2‐propanol controls differ from those of the ABVHRs (see Table 1). However, these concentrations are consistent with those found in most commercially available ABHSs and align with WHO recommendations for ethanol concentration [36] (these solutions are listed in the RKI list as ‘standard approvals’) (in German: Standardzulassungen) of the Federal Institute for Drugs and Medical Devices [37, 38]. ABVHRs and controls were provided by the hospital pharmacy of Bergmannsheil, Bochum, Germany.

#### 2.2.2 Pilot Study

In the pilot study we developed the procedures by modifying the protocol used by Löffler et al. [8] and incorporated repetitive patch testing [39] for two consecutive days with an alcohol‐containing solution. Fifteen participants (*N* = 15) were tested on the inner parts of both forearms. In the pilot study, we examined the differences between various pure alcoholic solutions used in ABHS: 60% and 90% ethanol, 20% and 40% 1‐propanol, and 40% and 70% 2‐propanol. We also included water and 0.7% PA g/g in water as controls. For detailed results, please refer to the Supporting Information (Table S2 and Figure S1).

#### 2.2.3 Main Study

**FIGURE 1:**
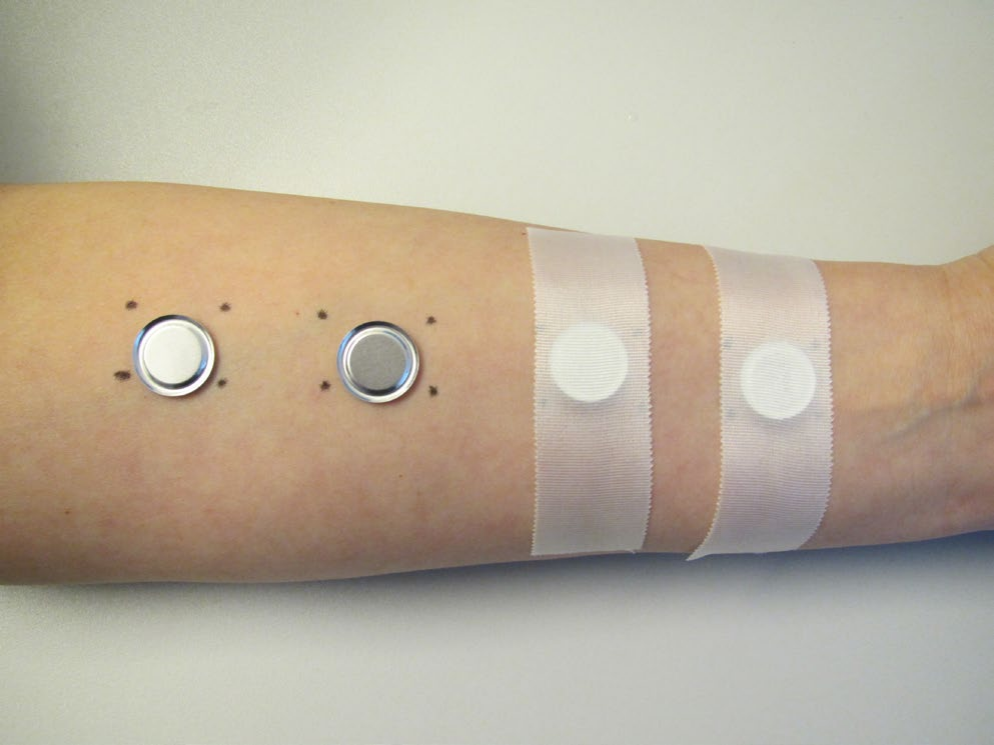
Application of patch‐chambers on forearm after randomisation.

**FIGURE 2:**
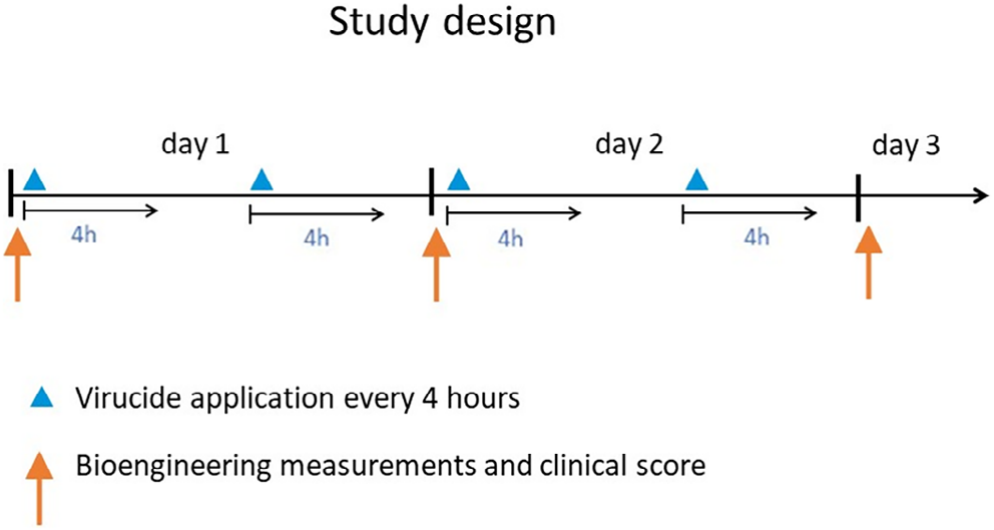
Study design. Blue triangles indicate patch testing with AHS and controls for 4 h applied twice daily. Orange arrows mark the time of measurements.

Test areas on both inner parts of forearms were marked (Figure 1). The ABVHRs and controls were assigned letters from A to H, and 50 μL of each solution was applied, single‐blinded for participants, twice daily for four hours under patch occlusion, with a pause between applications of 2 h for two consecutive days (Figure 2). We used large Finn Chambers (diameter 12 mm); Epitest Ltd. Oy, Tuusula, Finland, which were fixed with adhesive tape (Scanpor). The selected areas on the forearms were, as per the study protocol, exposed in cycle (four areas on each forearm). The negative control was an area on the left upper arm (no patch occlusion was used).

#### 2.2.4 Visual Scoring

All evaluations were carried out by the same investigator. Clinical assessment was performed before the application on days 1 and 2 and after a 24‐h rest period on day 3. We calculated the cumulative irritation score as proposed by the Standardisation Group of the European Society of Contact Dermatitis [40]. Each criterion, such as erythema, oedema, dryness, scaling, and fissuring (0/0.5/1/2/3), was rated separately, and the resulting total score was used for final analysis.

#### 2.2.5 Atopic Skin Diathesis

Atopic skin diathesis (ASD) was assessed by means of the Erlangen atopy score (Diepgen and Fartasch) [41, 42]. Serological parameters like total IgE and specific IgE and/or prick test for environmental allergens were not assessed. ASD was considered to be positive with a score of ≥ 10, and doubtful with a score of 8 to 9.

#### 2.2.6 Bioengineering Measurements

Measurements were taken on the first day (baseline, D1) before the start of the study, on day two (D2) and on day three (D3).

All measurements were performed in a climate‐controlled laboratory [43]. Participants rested in the laboratory for 20 min. Skin temperature was measured prior to transepidermal water loss (TEWL) (g/m^2^/h) with the Derma Temp DT 1001 RS (temperature range, 18°C–33°C); (Exergen, Watertown, MA, USA).

To ascertain TEWL, we obtained two measurements for each area and arithmetic means were used for further analysis. MDD4/Tewameter TM 300; Courage & Khazaka, Cologne, Germany assessed TEWL. Skin capacitance (corneometry) measuring stratum corneum hydration was assessed by Corneometer CM 820, PH900/SM810 (Courage and Khazaka) (measuring surface 49 mm^2^; frequency, 0.9–1.2 MHz; accuracy, ± 3%) and expressed in arbitrary units (AU, 0–99). We used chromametry, measuring skin reflectance (Chromameter CR 300; Konica Minolta Photo Imaging Europe, Langenhagen, Germany), to assess erythema in accordance with the *Commission Internationale de l'Eclairage* (CIE). Redness relates to the inflammation and is expressed by a* colour coordinates. All measurements were conducted in accordance with guidelines [43, 44, 45, 46].

### 2.3 Statistics

Statistical analysis was conducted in cooperation with the Department of Occupational Dermatology, Ruprecht‐Karls University, Heidelberg, Germany. The statistical analysis was performed with SPSS, version 27, and R statistical package (https://www.r‐project.org/). Graphs were designed on GraphPad Prism, version 10.2.3 (GraphPad Software Inc., La Jolla, CA).

Sample size was calculated with G*Power 3.1 software [47], assuming two‐tailed alternative hypothesis testing with a statistical power of 90% and an alpha level of 0.05. Identifying, at a minimum, a medium effect size between two dependent groups using the Wilcoxon signed‐rank‐sum test for matched pairs requires a sample size of *n* = 46.

The primary endpoint was to identify differences in change between baseline and after exposure at day 3 (Δ D3–D1) for TEWL as well as colorimetry and corneometry between the four virucidal products. The secondary endpoint was to evaluate Δ D3–D1 between virucidal products and the controls including water. The values are shown as means with standard deviations (SD) and presented in box plots with the median. The bottom line of the box represents the fifth percentile (Q1), and the top line represents the ninety‐fifth percentile (Q3). Outliers are plotted as asterisks (beyond the lower and upper limits). We applied the Benjamini‐Hochberg [48] procedure to adjust the *P*‐values and decrease the false discovery rate in multiple tests for the primary and secondary endpoints.

## 3 Results

### 3.1 Subject and Environmental Characteristics

In total, 48 healthy Caucasian volunteers met the inclusion criteria, signed a written consent and finalised all study procedures. The mean age was 37.4 years (range 18–61) and 32% of the subjects were females (Table 2). Almost 28% of participants had ASD (Erlanger atopy score ≥ 10). There were no significant differences between sexes with regard to age or ASD.

**TABLE 2:** Study population and study conditions.

	Males	Females	Total
Sex (*N* (%))	16 (33.3)	32 (66.7)	48 (100)
Age in years (mean (SD))	35.6 (14.9)	38.8 (12.5)	37.4 (13.3)
Erlangen atopy score (mean, (SD))	6.5 (5.5)	8.2 (3.8)	7.6 (4.5)
< 8 (*N* (%))	9 (64.3)	12 (41.4)	21 (48.8)
8–9 (*N* (%))	3 (21.4)	7 (24.1)	10 (23.2)
≥ 10 (*N* (%))	2 (14.3)	10 (34.5)	12 (27.9)
Study conditions (mean (SD))^a^			
Skin temperature in °C D1	31.2 (0.8)	30.7 (0.8)	30.9 (0.9)
Skin temperature in °C D3	30.3 (2.7)	30.5 (0.9)	30.4 (1.7)
Room temperature in °C D1	20.9 (0.6)	20.8 (0.6)	20.9 (0.55)
Room temperature in °C D3	21.6 (2.7)	20.8 (0.6)	21.1 (1.6)
Humidity D1	41.4 (8.9)	42.6 (10.8)	42.2 (10.1)
Humidity D3	42.5 (9.9)	42.7 (9.5)	42.6 (9.5)

*Note*: Table 2 presents the basic parameters of the study population (including Erlangen atopy score values, *n* = 5 missing) differentiated by sex and study conditions on day 1 (D1) and day 3 (D3).

Abbreviation: SD, standard deviation.

^a^ No significant variation between the days.

The study conditions were similar with regard to room, skin temperature, and humidity on day three (Table 2). The room temperature on day 1 was 20.9°C and the humidity was 42.2%; those values did not change (no statistically significant changes) between day 1 and day 3. The mean skin temperature on day one was 30.9°C (SD 0.87).

### 3.2 Bioengineering Measurements

No statistically significant changes were observed in the negative control on the left upper arm across any of the bioengineering measurements. The mean TEWL was 6.95 (SD 1.53) on day 1 and 6.93 (SD 1.45) on day 3. The mean corneometry values were 32.98 (SD 8.91) on day 1 and 31.53 (SD 7.2) on day 3. Similarly, the mean colorimetry*a was 7.6 (SD 1.38) on day 1 and 7.53 (SD 1.44) on day 3.

A Wilcoxon rank‐sum test revealed significant differences between all mean values for the tested solutions between baseline assessment at day 1 (D1) and assessment after exposure at day 3 (D3) for TEWL, corneometry (except for water) and colorimetry (except for water and PA) (Table 3). The greatest increase in TEWL was found in ABVHR‐D with a mean at day 1 of 6.43 (SD 1.40) to 8.76 (SD 3.87) at day 3 and, likewise, in corneometry, the most pronounced difference was seen in ABVHR‐D with a mean of 36.27 (SD 7.48) at day 1 to 24.13 (SD 5.82) at day 3. Finally, colorimetry * a revealed the greatest increase for ABVHR‐D. Further information about the changes on day 2 is provided in the supplement (Table S1).

**TABLE 3:** Bioengineering parameters for all substances at baseline (day 1) and after exposure at day 3.

Measurement	Group	Day 1	Day 3	*p*	Adjusted
		Mean (SD)	Mean (SD)		*p*‐Value
TEWL (g/m^2^/h)	Water	6.42 (1.42)	7.63 (1.99)	< 0.0001	< 0.0024
	Isopropanol	6.48 (1.49)	8.12 (2.29)	< 0.0001	< 0.0024
	Ethanol	6.39 (1.47)	7.98 (2.18)	< 0.0001	< 0.0024
	Phosphoric acid	6.37 (1.52)	7.82 (2.01)	< 0.0001	< 0.0024
	A	6.46 (1.44)	8.32 (2.37)	< 0.0001	< 0.0024
	B	6.44 (1.40)	7.91 (1.97)	< 0.0001	< 0.0024
	C	6.46 (1.43)	8.03 (2.29)	< 0.0001	< 0.0024
	D	6.43 (1.40)	8.76 (3.87)	< 0.0001	< 0.0024
Corneometry (AU)	Water	36.04 (9.21)	36.73 (6.95)	< 0.4270	< 0.4407
	Isopropanol	36.17 (8.97)	24.60 (5.09)	< 0.0001	< 0.0024
	Ethanol	36.19 (9.24)	24.54 (4.79)	< 0.0001	< 0.0024
	Phosphoric acid	35.69 (7.90)	31.60 (6.93)	< 0.0001	< 0.0024
	A	36.00 (9.04)	24.88 (5.05)	< 0.0001	< 0.0024
	B	35.35 (7.96)	25.27 (6.05)	< 0.0001	< 0.0024
	C	36.56 (9.20)	29.35 (4.97)	< 0.0001	< 0.0024
	D	36.27 (7.48)	24.13 (5.82)	< 0.0001	< 0.0024
Colorimetry * a	Water	8.03 (1.47)	8.03 (1.48)	< 0.7197	< 0.7197
	Isopropanol	8.06 (1.49)	8.77 (1.56)	< 0.0018	< 0.0019
	Ethanol	7.72 (1.20)	9.08 (1.98)	< 0.0001	< 0.0024
	Phosphoric acid	7.74 (1.47)	7.98 (1.46)	< 0.1876	< 0.2001
	A	8.01 (1.29)	8.66 (1.51)	< 0.0066	< 0.0073
	B	7.68 (1.52)	8.42 (1.74)	< 0.0014	< 0.0015
	C	7.91 (1.41)	8.90 (1.70)	< 0.0001	< 0.0024
	D	7.92 (1.55)	9.84 (2.29)	< 0.0001	< 0.0024

*Note*: Table 3 shows the absolute values for bioengineering measurements on day 1 and day 3. *p*‐values were calculated using the Wilcoxon signed‐rank test and adjusted using the Benjamini‐Hochberg method, comparing each substance to itself between day 1 and day 3.

Abbreviations: ABVHR A‐D, controls (water, 70% isopropanol, 80% ethanol, phosphoric acid 0.7%); SD, standard deviation; TEWL, transepidermal water loss.

### 3.3 Comparison of ABVHRs and ABVHRs With the Controls (Δ D3 to D1)

#### 3.3.1 TEWL (g/m^2^/h)

**FIGURE 3:**
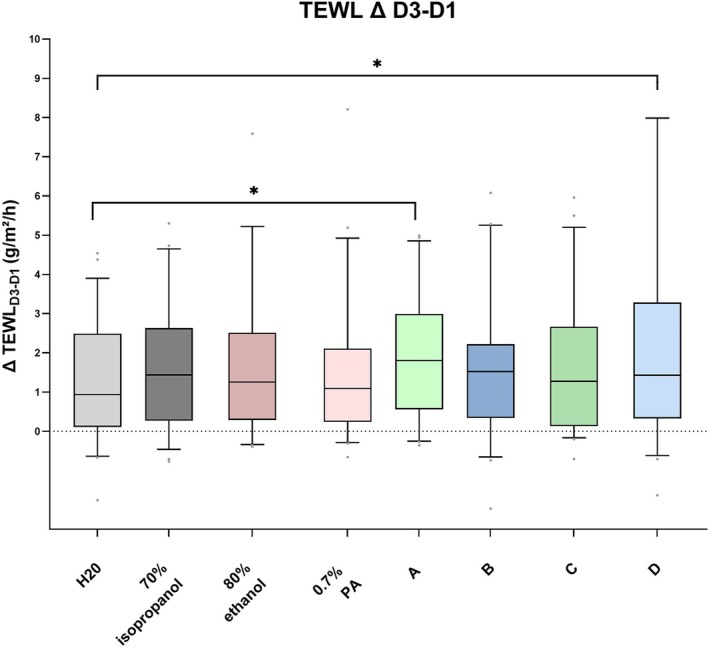
Differences D3–D1 for transepidermal water loss (TEWL). Graph depicting boxplots with median values and whiskers showing 5th–95th percentiles for TEWL value differences between D3 and D1. Statistically significant differences for the secondary endpoint are indicated by an asterisk (adjusted *p* < 0.05).

Only minor differences of TEWL values between the tested solutions were revealed. Although Wilcoxon signed rank sum test comparing ABVHR‐A with ABVHR‐D showed no significant differences (adjusted *p* > 0.05), these ABVHRs showed the most pronounced differences (Table 4). Even though differences between the ABVHRs were not statistically significant, ABVHR‐A and ABVHR‐D showed a significant increase of TEWL as compared to water (adjusted *p* < 0.05; Figure 3, Table 4).

**TABLE 4:** Mean change between day 3 (D3) and baseline (D1) in bioengineering parameters for each substance.

Measurement	Group	Δ D3‐D1	Primary endpoint^a^	Secondary endpoint^b^
		Mean (SD)		
TEWL (g/m^2^/h)	Water	1.21 (0.21)		A, D
	Isopropanol	1.64 (0.22)		
	Ethanol	1.59 (0.24)		
	Phosphoric acid	1.45 (0.24)		
	A	1.86 (0.22)		Water
	B	1.46 (0.23)		
	C	1.56 (0.23)		
	D	2.33 (0.53)		Water
Corneometry (AU)	Water	0.71 (1.04)		A, B, C, D
	Isopropanol	−11.56 (1.23)		C
	Ethanol	−11.65 (1.03)		A, B, C
	Phosphoric acid	−4.08 (0.78)		A, B, C, D
	A	−11.13 (1.13)	C	Water, PA, Eth
	B	−10.08 (0.81)	C	Water, PA, Eth
	C	−7.21 (1.19)	A, B, D	Water, Iso, PA, Eth
	D	−12.15 (0.98)	C	Water, PA
Colorimetry * a	Water	−0.01 (0.13)		A, B, C, D
	Isopropanol	0.70 (0.21)		D
	Ethanol	1.36 (0.25)		A, B
	Phosphoric acid	0.24 (0.17)		C, D
	A	0.65 (0.21)	C, D	Water, Eth
	B	0.74 (0.21)	D	Water, Eth
	C	0.99 (0.21)	A, D	Water, PA
	D	1.91 (0.31)	A, B, C	Water, Iso, PA

Abbreviation: TEWL, transepidermal water loss.

^a^ The primary endpoint is defined as significant differences in change (Δ D3‐D1) comparing ABVHRs (A‐D). Only substances with an adjusted *p*‐value < 0.05 are shown.

^b^ Secondary endpoint defined as significant differences in change (Δ D3‐D1) between each virucidal product and controls: water, 70% isopropanol (Iso), 80% ethanol (Eth), 0.7% phosphoric acid (PA). Only substances with an adjusted *p*‐value < 0.05 are shown.

#### 3.3.2 Corneometry (AU)

Wilcoxon signed rank‐sum tests between ABVHR‐A to D and those between the ABVHRs and their controls revealed significant differences (adjusted *p* < 0.05). Skin hydration as measured by corneometry decreased in all alcohol‐containing solutions (mean change > 10 AU). With the exception of ABVHR‐C, these values were not significantly different between alcohol‐containing solutions. The mean difference from D3 to D1 for ABVHR‐C (−7.21 AU) was significantly lower compared to other alcohols and the other ABVHRs (Table 4) (even after *P*‐adjustment). The value for PA decreased (mean − 4.08 AU), the difference in change for PA was significantly smaller compared to ABVHR A, B, C, and D.

#### 3.3.3 Colorimetry*a (Erythema)

All alcohol‐containing solutions induced erythema, the highest increase of the colorimetry * a value was found in ABVHR‐D (mean change 1.91) which was significantly higher than all other tested alcohol‐containing solutions, including ABVHR A, B, and C, with the exception of 80% ethanol (mean change 1.36) (Table 4). All ABVHRs showed a significantly higher increase in colorimetry * a compared to water and PA (adjusted *p* < 0.05). PA increased erythema slightly and was similar in comparison with water.

### 3.4 Clinical Scoring and Atopic Skin Diathesis

**FIGURE 4:**
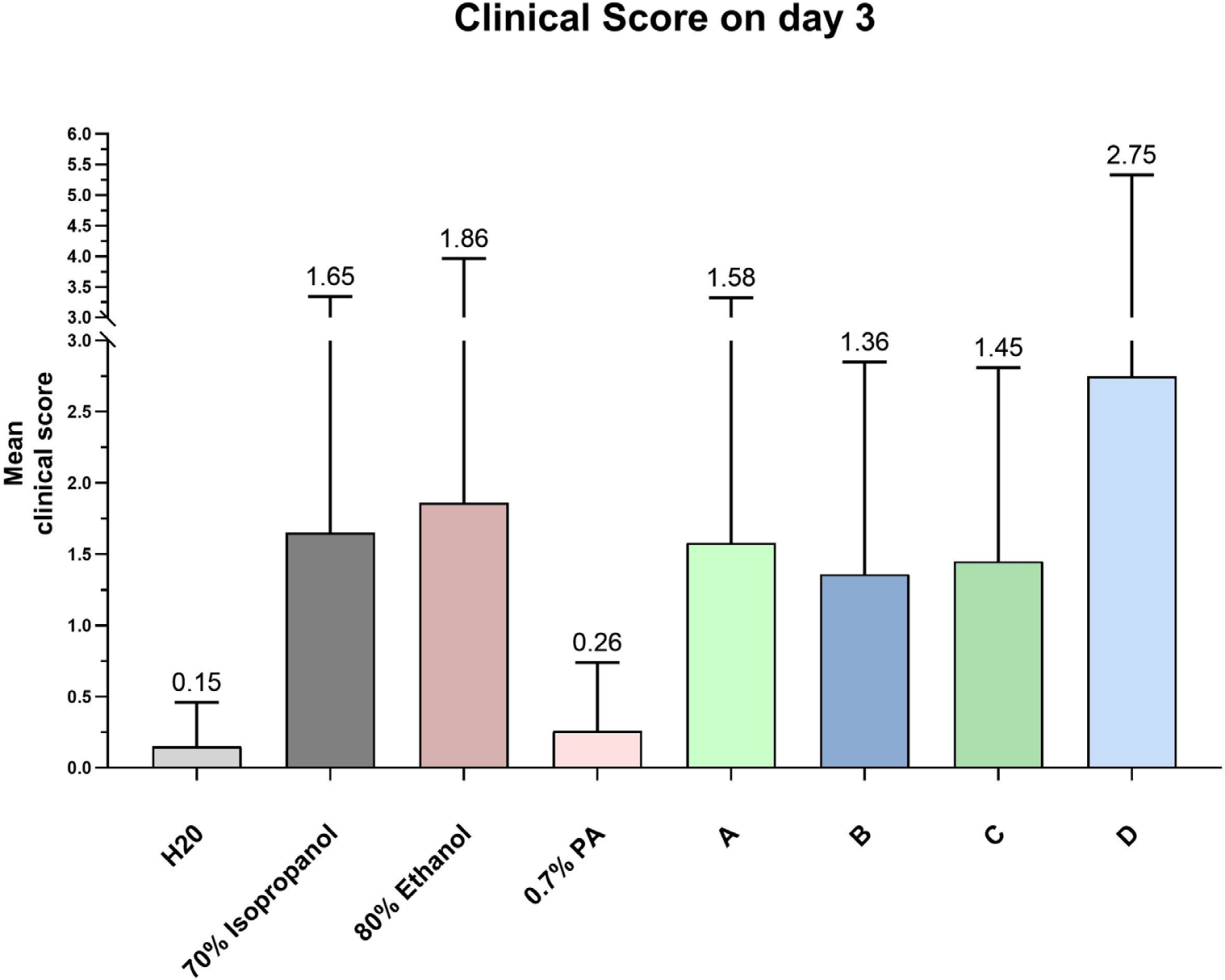
Clinical score values on day 3. Bar chart depicting the mean clinical scores on day 3, with whiskers representing the standard deviations.

The highest mean score value in clinical scoring on day 3 was recorded for ABVHR‐D (mean 2.8, SD 2.6), which was significantly higher than for ABVHR‐A (mean 1.6, SD 1.7), ABVHR‐B (mean 1.4, SD 1.5) and ABVHR‐C (mean 1.5, SD 1.4) (Figure 4). ABVHR A‐C revealed similar scores, which deviated not significantly from the corresponding alcohol‐containing controls (ethanol, 2‐propranol). The lowest clinical scores were recorded for PA and water.

In the subanalysis for atopic and non‐atopic individuals, there were no significant differences between these groups in either clinical scores or bioengineering values.

## 4 Discussion

The most important finding is that all the ABVHRs we tested caused skin irritation, as evidenced by bioengineering measurements and clinical scores. However, they differed in their potential to cause irritation. The most pronounced differences were observed in corneometry (decrease in skin hydration) and colorimetry. ABVHR‐D had the potential to evoke the most severe changes, indicating stronger irritation. Additionally, the ethanol and 2‐propanol controls showed similar or higher trends in skin irritation measurements compared to the other tested ABVHRs, with the exception of ABVHR‐D. Interestingly, ABVHR‐C had the best profile in corneometry but still caused a slight increase in erythema. These results correlated with clinical skin evaluations, where the highest values of irritation were caused by ABVHR‐D and alcohol‐containing controls, followed by the other ABVHRs. Importantly, our study found no evidence of increased irritation due to PA at the tested concentration.

Our findings support previous research that showed ABHRs labelled as having ‘virucidal activity’ can cause skin dehydration and an increase in erythema [10]. This might indicate the onset of inflammation, as changes were detected already one day after applying ABVHRs. Since this reaction was delayed, it seems unlikely that the redness was due to reflex vasodilation.

The irritation caused by ABVHR‐D was the most severe among the tested solutions. In contrast, ABVHR‐C, which also contained PA, exhibited a much milder profile. The key differences between these two solutions were the lower concentration of 1‐propanol in ABVHR‐C (10 g versus 18 g per 100 g) and the presence of additives, although the concentration of glycerol was not provided (see Table 1). One possible explanation for the difference in irritation levels is the use of different non‐volatile ingredients or moisturisers, although the impact of 1‐propanol is also likely. In our pilot study using the same repetitive occlusion model (see Supporting Information), we observed statistically significant changes in TEWL between days one and three for 1‐propanol at concentrations of 20% and 40% (analogous to Table 3).

Previous research also found that alcohols varied in their irritative potential [8, 48]. A recent review on the impact of propanol on skin barrier function concluded that 1‐propanol can cause irritation, especially at concentrations above 60% [49]. However, our results (pilot study) align with other studies [13] which suggest that such irritation can also occur at much lower concentrations. This effect could be intensified by preceding low‐grade trauma from occlusion/water [13, 50] or, in our study, by patch occlusion. However, in comparison to ethanol, the irritative potential of 1‐propanol must be weighed against its superior bactericidal activity, allowing lower total concentrations when used alone or in combination with other alcohols [51]. The biological properties of ABHRs can vary depending on the percentages of alcohols used, and this cannot be determined by examining the individual components in isolation. To our knowledge, no published studies have investigated the impact of ABHRs with different compositions (proportion of ethanol to propanol) on skin barrier function. However, to date, there is no clear epidemiological evidence for increased skin irritation from such products. Further research is needed to find the optimal balance between alcohol concentration, ABHRs composition, biocidal activity, and skin irritation.

Using corneometry, Löffler et al. found that the biological response of the skin varies depending on the concentration of alcohol [8]. In their study, ethanol and 1‐ and 2‐propanol at lower concentrations (all above 60%) caused more skin dehydration than their higher‐concentration counterparts. Soltanipoor et al. also observed a pronounced effect of 60% 1‐propanol on skin hydratation [52]. However, the impact of 1‐propanol on skin hydration might be different in individuals with atopic skin, who experience less dehydration but have higher visual scores and higher TEWL values compared to healthy subjects, indicating the development of skin irritation [13].

In a subgroup analysis of individuals with and without ASD (Erlangen atopy score of ≥ 10 points), possibly due to the short duration of the study or low statistical power, we did not observe changes in the assessed skin parameters. Nonetheless, individuals with active atopic dermatitis (AD) were excluded from participating in the study. It is likely that active atopic dermatitis is a significant factor in increasing the susceptibility of the skin to irritation from ABHS [53, 54]. In the study by Angelova‐Fischer et al., individuals with atopic skin but without active AD also showed increased susceptibility to skin irritation [13]. This difference might be due to variations in the study protocol and criteria for atopy.

Acidic components can enhance the virus‐inactivating ability of ABVHRs [55], but there have been concerns about PA potentially damaging the skin barrier [23]. For example, the product “Manusept virucide” was withdrawn from the market by its manufacturer due to reported high rates of skin irritation following its use [23]. However, the procedure used in this study was markedly different from how hand sanitizers are used in real life.

In our study, PA, at the tested concentration, was well‐tolerated, showing effects similar to water, and did not appear to exacerbate irritation when used in conjunction with ABHRs. However, due to potential cofactors in our study, further research is necessary to validate these findings. Nevertheless, our findings align with those of Conrad et al. [10], who studied similar ABVHRs, including PA systems. In their study, P1 had alcohol concentrations comparable to ABVHR‐D, P2 to ABVHR‐A, and P3 to ABVHR‐C. They found that both P2 and P3 exhibited lower TEWL values compared to P1 and the reference. According to the authors, the only difference between P1 and the reference was the presence of PA, and the irritative potential of these substances was similar. This suggests that the inclusion of PA in ABVHRs did not increase skin irritation, indicating that PA has no significant impact on skin function. However, other factors might be involved, as some PA‐containing ABHRs have shown increased adverse events. PA was included in ABVHR‐C and D, with ABVHR‐C showing the best profile in terms of skin irritation compared to other tested alcoholic solutions. Hence, PA might be advantageous as it increases virucidal efficacy, potentially allowing reduced amounts of alcohols to be used while achieving the same effect. Additionally, the use of PA might improve employee acceptance, since it could represent a way to reduce the recommended application time (e.g., 2 min for ABVHR‐A and B vs. 1 min for ABVHR‐C and D). One could speculate that lactic acid, used in ABVHR‐B, also did not seem to increase the irritative properties of this solution.

Interestingly, in general, the ethanol control, and to a lesser extent the isopropanol control, showed a trend towards higher bioengineering values compared to the ABVHRs we tested (with the exception of ABVHR‐D), even though the concentrations of alcohol in ABVHR‐A and B were higher than in their ethanol counterpart. This could be due to the fact that alcohols at lower concentrations seem to have different effects on the skin, particularly in terms of skin hydration [8]. Additionally, the inclusion of additives and moisturising ingredients like propylene glycol, lanolin, and glycerin in the sanitizers could have played a beneficial role in improving skin hydration and reducing irritation. Glycerin, in particular, has been found to lessen irritation caused by ABHRs [56, 57] which is why it is used in the World Health Organisation's (WHO) hand rub formulation [58]. However, it is important to be aware of the concentration of glycerin, as it can affect the antimicrobial properties of ABHRs [59]. Therefore, a lower concentration of glycerin might be preferable, especially in tropical climates [58].

Although ABHRs can negatively affect the skin barrier function, they are less harmful than washing hands with detergent [60]. In clinical settings, it is recommended to replace hand washing with detergents by application of ABHRs whenever possible. Hand washing should be reserved for instances of visible soiling. Furthermore, experimental studies have shown that alternating the use of alcohols and detergent is more beneficial than using detergent alone [50, 60]. In some studies, alcohol can even lessen the irritation caused by a detergent [8, 61]. Therefore, if a less irritating ABHR with moisturisers is available, it should be particularly recommended for people with sensitive or atopic skin, or for those with defects of their skin barrier.

The study is limited due to its experimental design involving only healthy Caucasian individuals without a history of previous or ongoing skin diseases. The study design encompasses ABVHRs exposure for two days. As such, it measures only acute changes and is unable to evaluate long‐term use of such ABVHRs. In addition, their negative effect on the skin barrier and on the dermis resulting in erythema may be exacerbated by patch occlusion. However, even in practice, the use of ABHRs is often accompanied by direct glove occlusion (the use of gloves on wet hands is not recommended, but in our experience is common). Measuring the effects of ABVHRs without occlusion would require repeated exposures of more than 20 times per day, similar to the study by Yüksel et al. [62]. Furthermore, we restricted our alcoholic controls to 80% ethanol and 70% 2‐propanol, as these concentrations are commonly used in most ABHRs. However, we did not include 1‐propanol, which was only tested in the pilot study and is an alcoholic ingredient in some tested ABVHRs. Our experimental study had a relatively high sample size. However, its statistical power might be too small to detect some effects, resulting in a type‐B error. Nevertheless, to our knowledge, this is the first bioengineering study evaluating the role of PA as well as different ABVHRs formulations.

## 5 Conclusions

All the ABVHRs we tested caused skin irritation, as indicated by bioengineering measurements, but they varied in their potential to evoke irritation. This variation is probably attributable to the concentration of 1‐propanol (n‐propanol) in the ABVHRs and to the proportions and concentrations of alcohols or alcohol mixtures used. As a result, ABVHRs can have different biological effects on the skin. PA, at the concentration tested, seems to be well tolerated and could be a viable option to enhance the antimicrobial activity of ABVHRs. Consequently, the use of PA could enable reduced quantities of alcohol to be used while also achieving the desired virucidal effect.

## Author Contributions

**Michal Gina:** conceptualization, investigation, writing – original draft, methodology, validation, visualization, writing – review and editing, formal analysis, data curation, software. **Robert Ofenloch:** writing – original draft, visualization, writing – review and editing, software, formal analysis, data curation. **Ingeborg Schwebke:** methodology, writing – review and editing. **Nils‐Olaf Hübner:** methodology, writing – review and editing. **Thomas Brüning:** writing – review and editing. **Manigé Fartasch:** conceptualization, investigation, funding acquisition, writing – original draft, methodology, writing – review and editing, project administration, supervision, resources, validation.

## Conflicts of Interest

The authors declare no conflicts of interest.

## Supporting information


**Figure S1a.** Pilot Study: showing Boxplots for Changes in TEWL (Δ D3‐D1).

**Figure S1b.** Pilot Study: showing mean of clinical score on day 3.

Table S1‐S2

## Data Availability

The data that support the findings of this study are available on request from the corresponding author. The data are not publicly available due to privacy or ethical restrictions.
