# Supplementary figures and images for: The Effect of Alcohol‐Based Virucidal Hand Sanitizers on Skin Barrier Function—A Randomised Experimental Study
Source: Contact Dermatitis. 2025 May 12;93(2):119–30. doi: 10.1111/cod.14808 (PMC12223927; doi:10.1111/cod.14808)

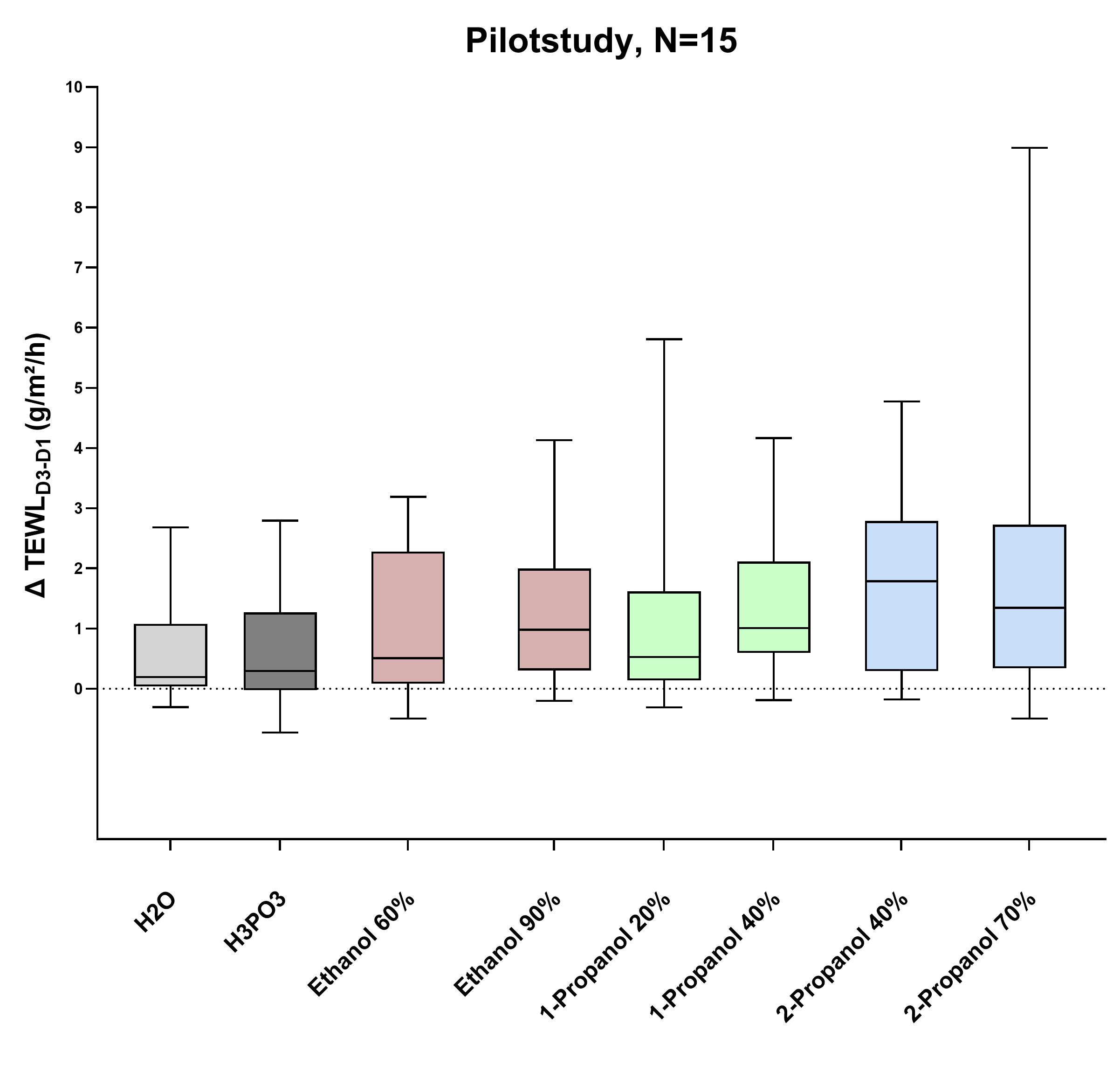

Supplement: Supplementary file 1 — Figure S1a. Pilot Study: showing Boxplots for Changes in TEWL (Δ D3‐D1). [file COD-93-119-s003.jpg]

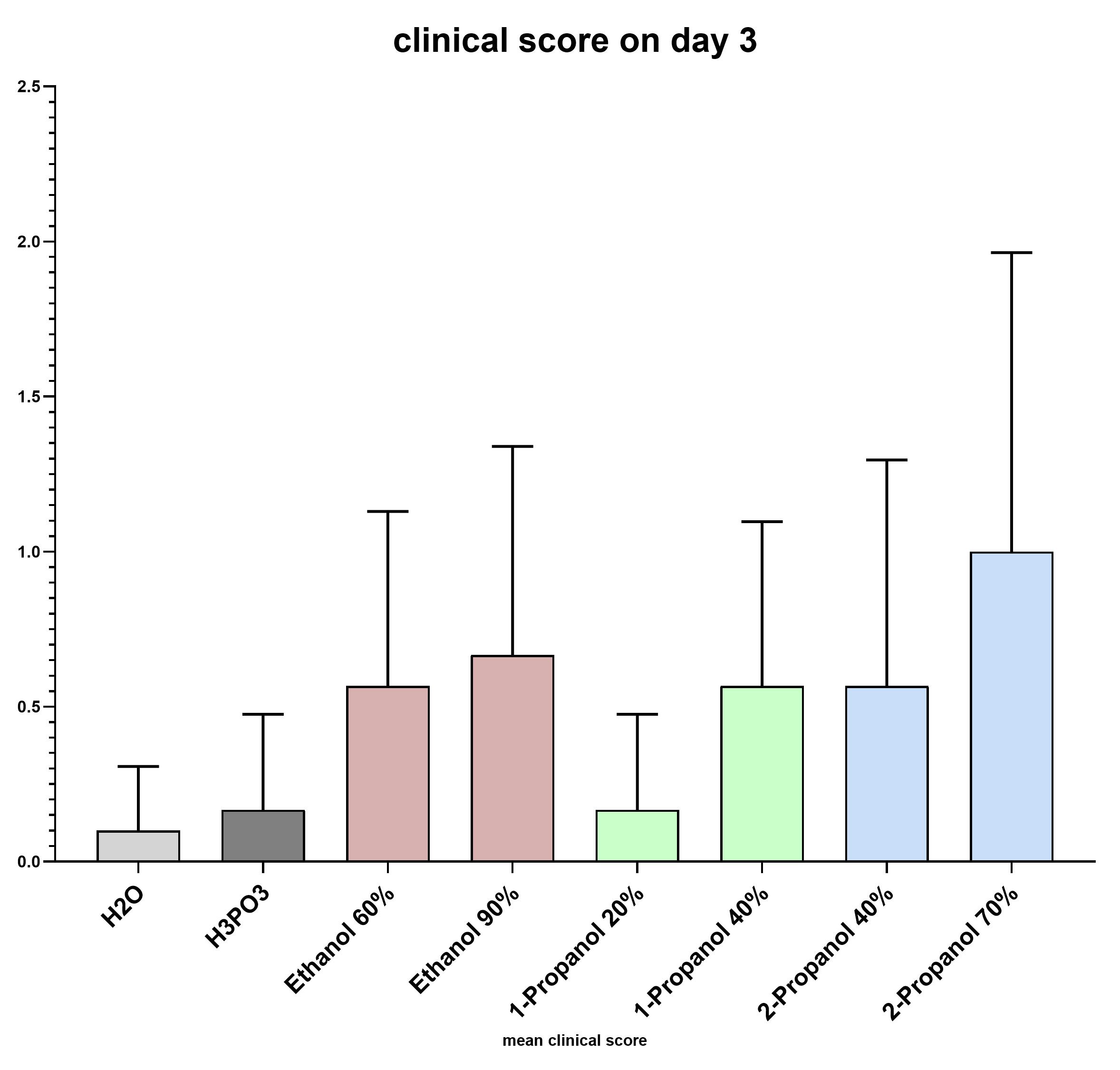

Supplement: Supplementary file 2 — Figure S1b. Pilot Study: showing mean of clinical score on day 3. [file COD-93-119-s002.jpg]
