# Supplementary material for: The Effect of Alcohol‐Based Virucidal Hand Sanitizers on Skin Barrier Function—A Randomised Experimental Study
Source: Contact Dermatitis. 2025 May 12;93(2):119–30. doi: 10.1111/cod.14808 (PMC12223927; doi:10.1111/cod.14808)
Supplement: Supplementary file 3 — Table S1‐S2 [file COD-93-119-s001.docx]

Supplement Table S1: Mean change of assessed bioengineering parameters between visits for all tested substances

|  | | H_2_0 | | 70%  2-propanol | | 80% ethanol | | 0.7% PA | | A | | B | | C | | D | |
| --- | --- | --- | --- | --- | --- | --- | --- | --- | --- | --- | --- | --- | --- | --- | --- | --- | --- |
|  |  | **mean** | SD | **mean** | SD | **mean** | SD | **mean** | SD | **mean** | SD | **mean** | SD | **mean** | SD | **mean** | SD |
| TEWL^#^ | Δ 1-2 | **1.29** | 0.19 | **1.45** | 0.21 | **1.45** | 0.21 | **1.14** | 0.19 | **1.36** | 0.22 | **1.30** | 0.21 | **1.44** | 0.24 | **1.58** | 0.25 |
|  | Δ 1-3 | **1.21**  a,d | 0.21 | **1.64** | 0.22 | **1.59** | 0.24 | **1.45** | 0.24 | **1.86** | 0.22 | **1.46** | 0.23 | **1.56** | 0.23 | **2.33** | 0.53 |
| Corneometry | Δ 1-2 | **-1.79**  a,b,c,d | 1.22 | **-9.46**  c | 1.08 | **-9.44**  **c** | 1.09 | **-5.25**  a,b,d | 0.71 | **-8.90**  c | 0.88 | **-7.35**  d | 0.81 | **-5.63**  a,d | 1.06 | **-9.98**  b,c | 1.00 |
|  | Δ 1-3 | **0.71**  a,b,c,d | 1.04 | **-11.56**  c | 1.23 | **-11.65**  c | 1.03 | **-4.08**  a,b,c,d | 0.78 | **-11.13**  c | 1.13 | **-10.08**  c,d | 0.81 | **-7.21**  a,b,d | 1.19 | **-12.15**  b,c | 0.98 |
| Colorimetry*a | Δ 1-2 | **0.13**  c,d | 0.12 | **0.49** | 0.14 | **0.94**  a,b | 0.16 | **0.15**  c,d | 0.11 | **0.30**  d | 0.15 | **0.36**  d | 0.13 | **0.58** | 0.16 | **0.98**  a,b | 0.26 |
|  | Δ 1-3 | **-0.01**  a,b,c,d | 0.13 | **0.70**  d | 0.21 | **1.36**  a,b,c | 0.25 | **0.24**  a,b,c,d | 0.17 | **0.65**  c,d | 0.21 | **0.74**  d | 0.21 | **0.99**  a,d | 0.21 | **1.91**  a,b,c | 0.31 |
| clinical score | Δ 1-2 | **0.11**  a,b,c,d | 0.04 | **1.02**  a | 0.15 | **0.95** | 0.16 | **0.25**  a,b,c,d | 0.05 | **0.63**  d | 0.11 | **0.68**  d | 0.12 | **0.76** | 0.11 | **1.19**  a,b | 0.22 |
|  | Δ 1-3 | **0.15**  a,b,c,d | 0.04 | **1.65**  d | 0.24 | **1.89** | 0.31 | **0.26**  a,b,c,d | 0.07 | **1.58**  d | 0.25 | **1.36**  d | 0.22 | **1.45**  d | 0.20 | **2.75**  a,b,c | 0.37 |

^#^TEWL (g/m^2^/h): transepidermal water loss; 0.7% phosphoric acid (PA); a significant difference (*P* < .05) in the change between the respective substance and ABVHR A-D is indicated by small letters under the mean values.

**Table S2.** **Descriptive Statistics from the Pilot Study (15 participants) for Changes in bioengineering parameters (Δ D3-D1)**

A) **Pilot Study: descriptive statistics for changes in TEWL (Δ D3-D1) for alcoholic solutions**

| variables | mean | sd | se_mean | IQR | p25 | p50 | p75 |
| --- | --- | --- | --- | --- | --- | --- | --- |
| H2O | 0.63 | 0.96 | 0.24 | 0.83 | 0.04 | 0.19 | 0.87 |
| PA | 0.59 | 0.99 | 0.25 | 0.99 | -0.007 | 0.29 | 0.98 |
| Ethanol_60% | 1.18 | 1.25 | 0.32 | 2.02 | 0.14 | 0.50 | 2.16 |
| Ethanol_90% | 1.2 | 1.13 | 0.29 | 1.55 | 0.31 | 0.98 | 1.87 |
| 1_Propanol_20% | 1.05 | 1.52 | 0.39 | 1.26 | 0.21 | 0.52 | 1.47 |
| 1_Propanol_40% | 1.35 | 1.10 | 0.28 | 1.45 | 0.63 | 1.00 | 2.08 |
| 2_Propanol_40% | 1.81 | 1.53 | 0.39 | 2.29 | 0.45 | 1.78 | 2.74 |
| 2_Propanol_70% | 1.92 | 2.26 | 0.58 | 2.06 | 0.65 | 1.34 | 2.71 |

b) **Pilot Study: descriptive statistics for changes in corneometry (Δ D3-D1) for alcoholic solutions**

| variables | mean | sd | se_mean | IQR | p25 | p50 | p75 |
| --- | --- | --- | --- | --- | --- | --- | --- |
| H2O | 0.8 | 3.68 | 0.95 | 6.0 | -2.5 | 0 | 3.5 |
| PA | -4.06 | 4.25 | 1.09 | 4.5 | -6.5 | -5 | -2.0 |
| Ethanol_60% | -9.86 | 4.64 | 1.19 | 5.5 | -12.5 | -10 | -7.0 |
| Ethanol_90% | -10.46 | 5.48 | 1.41 | 7.0 | -14.5 | -10 | -7.5 |
| 1_Propanol_20% | -4.8 | 4.19 | 1.08 | 3.5 | -6.5 | -4 | -3.0 |
| 1_Propanol_40% | -9.6 | 3.94 | 1.01 | 4.5 | -11.0 | -10 | -6.5 |
| 2_Propanol_40% | -8.66 | 4.99 | 1.28 | 7.0 | -13.0 | -7 | -6.0 |
| 2_Propanol_70% | -11.26 | 4.72 | 1.22 | 7.5 | -15.0 | -12 | -7.5 |

c) **Pilot Study: descriptive statistics for changes in colorimetry*a (Δ D3-D1) for alcoholic solutions**

| variables | mean | sd | se_mean | IQR | p25 | p50 | p75 |
| --- | --- | --- | --- | --- | --- | --- | --- |
| H2O | 0.41 | 1.3 | 0.33 | 1.53 | -0.28 | 0.37 | 1.25 |
| PA | 0.04 | 1.83 | 0.47 | 1.15 | -0.45 | 0.04 | 0.7 |
| Ethanol_60% | 1.56 | 2.08 | 0.53 | 1 | 0.80 | 1.09 | 1.81 |
| Ethanol_90% | 0.99 | 0.99 | 0.25 | 1.24 | 0.35 | 0.91 | 1.59 |
| 1_Propanol_20% | 0.38 | 1.21 | 0.31 | 1.44 | -0.10 | 0.51 | 1.33 |
| 1_Propanol_40% | 0.46 | 0.97 | 0.25 | 0.99 | -0.09 | 0.29 | 0.89 |
| 2_Propanol_40% | 1.50 | 1.44 | 0.37 | 1.6 | 0.62 | 1.19 | 2.22 |
| 2_Propanol_70% | 1.29 | 1.47 | 0.38 | 1.5 | 0.50 | 0.88 | 2.01 |

**Table S2:** Descriptive Statistics from the Pilot Study for Changes in bioengineering parameters (Δ D3-D1): a) TEWL b) corneometry c) colorimetry*a. PA- phosphoric acid, IQR – interquartile range (p25-p75); SD – standard deviation; se_mean – standard error of the mean.
